# Maternal urinary concentrations of organophosphate ester metabolites: associations with gestational weight gain, early life anthropometry, and infant eating behaviors among mothers-infant pairs in Rhode Island

**DOI:** 10.1186/s12940-020-00648-0

**Published:** 2020-09-11

**Authors:** Kathryn A. Crawford, Nicola Hawley, Antonia M. Calafat, Nayana K. Jayatilaka, Rosemary J. Froehlich, Phinnara Has, Lisa G. Gallagher, David A. Savitz, Joseph M. Braun, Erika F. Werner, Megan E. Romano

**Affiliations:** 1 Department of Epidemiology, Geisel School of Medicine at Dartmouth, Lebanon, NH USA; 2 Current Address: Program in Environmental Studies, Middlebury College, Middlebury, VT USA; 3 Department of Epidemiology, Yale University School of Public Health, New Haven, CT USA; 4 Division of Laboratory Sciences, National Center for Environmental Health, Centers for Disease Control and Prevention, Atlanta, GA USA; 5 Department of Obstetrics, Gynecology, and Reproductive Sciences, University of Pittsburgh, Pittsburgh, PA USA; 6 Division of Maternal-Fetal Medicine, Women & Infants Hospital of Rhode Island, Providence, RI USA; 7 Department of Epidemiology, Brown University School of Public Health, Providence, RI USA; 8 Department of Obstetrics and Gynecology, Warren Alpert Medical School of Brown University, Providence, RI, USA

**Keywords:** Organophosphate ester (OPE), Triphenyl phosphate (TPHP), Tri-2-chloroethyl phosphate [TCEP], Tri (1,3-dichloro-2-propyl) phosphate [TDCPP], Pregnancy, Infant anthropometry, gestational weight gain (GWG), Baby eating behavior questionnaire (BEBQ)

## Abstract

**Background:**

Organophosphate esters (OPEs)—used as flame retardants and plasticizers—are associated with adverse pregnancy outcomes such as reduced fecundity and live births and increased preterm delivery. OPEs may interfere with growth and metabolism via endocrine-disruption, but few studies have investigated endocrine-related outcomes. The objective of this pilot study (*n* = 56 mother-infant pairs) was to evaluate associations of OPEs with gestational weight gain (GWG), gestational age at delivery, infant anthropometry, and infant feeding behaviors.

**Methods:**

We quantified OPE metabolites (bis-2-chloroethyl phosphate [BCEP], bis (1,3-dichloro-2-propyl) phosphate [BDCPP], diphenyl phosphate [DPHP]) in pooled maternal spot urine collected throughout pregnancy (~ 12, 28, and 35 weeks’ gestation). We obtained maternal sociodemographic characteristics from questionnaires administered at enrollment and perinatal characteristics from medical record abstraction. Trained research assistants measured infant weight, length, head and abdominal circumferences, and skinfold thicknesses at birth and 6 weeks postpartum. Mothers reported infant feeding behavior via the Baby Eating Behavior Questionnaire (BEBQ). Using multiple linear regression, we assessed associations of log_2_-transformed maternal urinary OPE metabolites with GWG, gestational age at delivery, infant anthropometry at birth, weekly growth rate, and BEBQ scores at 6 weeks postpartum. We used linear mixed effects (LME) models to analyze overall infant anthropometry during the first 6 weeks of life. Additionally, we considered effect modification by infant sex.

**Results:**

We observed weak positive associations between all OPE metabolites and GWG. In LME models, BDCPP was associated with increased infant length (β = 0.44 cm, 95%CI = 0.01, 0.87) and weight in males (β = 0.14 kg, 95%CI = 0.03, 0.24). BDCPP was also associated with increased food responsiveness (β = 0.23, 95%CI = 0.06, 0.40). DPHP was inversely associated with infant abdominal circumference (β = − 0.50 cm, 95%CI = − 0.86, − 0.14) and female weight (β = − 0.19 kg, 95%CI = − 0.36, − 0.02), but positively associated with weekly growth in iliac skinfold thickness (β = 0.10 mm/wk., 95%CI = 0.02, 0.19). Further, DPHP was weakly associated with increased feeding speed. BCEP was associated with greater infant thigh skinfold thickness (β = 0.34 mm, 95%CI = 0.16, 0.52) and subscapular skinfold thickness in males (β = 0.14 mm, 95%CI = 0.002, 0.28).

**Conclusions:**

Collectively, these findings suggest that select OPEs may affect infant anthropometry and feeding behavior, with the most compelling evidence for BDCPP and DPHP.

## Introduction

The use of organophosphate esters (OPEs) as flame retardants in consumer products such as residential and office furniture, baby products, and electronics has increased since polybrominated diphenyl ethers (PBDEs) were phased-out of use and production between 2004 and 2013 in the USA [1] amid growing concerns about their toxicity [2–5]. The most commonly used OPEs are not chemically bound to materials and can migrate from products over time [6]. As a result, OPEs are commonly found in indoor environments, including household dust [7–12]. OPEs are also used as plasticizers in common consumer products such as nail polish [13]. Human exposure to OPEs is understood to be ubiquitous and multiple studies have detected OPE metabolites in the urine of the majority of the general population in the United States [14–22] and around the world [23–26].

Experimental studies suggest that OPEs are endocrine-disrupting compounds that may interfere with growth and metabolism. In vitro, OPEs interfere with the estrogen receptors (ER*a* and ERß), androgen receptor (AR), glucocorticoid receptor (GR), pregnane X receptor (PXR), peroxisome proliferator-activated receptor gamma (PPARγ), and mineralocorticoid receptor (MR) [27–30], which, in turn, could potentially affect steroidogenesis, growth, development, and metabolic homeostasis (summarized in Dishaw et al. [31]). For example, the OPE triphenyl phosphate (TPHP) can alter levels of glucose, pyruvate, triglycerides, and cholesterol, and affect lipid homeostasis at molecular and phenotypic levels [32, 33]. Exposures occurring during early life appear to be more strongly associated with adverse endocrine and developmental effects than exposures occurring later in life. Notably, mice exposed to environmentally-relevant concentrations of OPEs during the perinatal period exhibited impaired weight gain and weight regulation throughout the life course, and molecular biomarkers of lipid dysregulation [34, 35]. Collectively, in vitro and in vivo toxicological studies provide evidence that OPEs have the capacity to disrupt growth and metabolism through endocrine-related mechanisms of action.

Epidemiologic studies of the endocrine-disrupting and reproductive effects of OPEs are limited but suggest that similar adverse effects of OPE exposure occur in humans as well. Certain OPEs have been associated with thyroid hormone disruption in women [36], and reduced sperm quality, reduced fertilization, and altered hormone levels in men [9, 37, 38]. Organophosphate esters were found to adversely affect pregnancy outcomes (e.g., successful fertilization, implantation, clinical pregnancy and live births) in a cohort of women undergoing in vitro fertilization [14]. Most recently, a study investigating gestation and fetal growth found an association between maternal urinary OPE metabolites and gestational age at delivery, with evidence of effect modification (EM) by infant sex [19].

Considering prior research, which suggests that OPEs may alter metabolic homeostasis and induce weight gain, we evaluated the hypotheses that OPE exposure during pregnancy affects: gestational weight gain (GWG) among pregnant women, infant gestational age at delivery, infant anthropometric measures of size and body composition at birth and growth during the first 6 weeks of life, and infant feeding behavior in a pregnancy cohort. To our knowledge, this is the first epidemiologic study to evaluate GWG and measures of newborn growth and feeding behavior in the context of prenatal OPE exposure.

## Methods

### Study setting and participants

Between July and December 2014, we enrolled 62 women from prenatal clinics affiliated with Women & Infants Hospital of Rhode Island (WIHRI), which provides care for approximately 80% of deliveries to state residents. Women were eligible for enrollment if they were ≥18 years old, ≤20 weeks gestation, English speaking residents of Rhode Island, and intended to deliver at WIHRI. Women were excluded if they had a multifetal pregnancy or had been diagnosed with or were currently receiving treatment for serious chronic health issues including, thyroid/renal disorders, HIV, cardiovascular disease other than hypertension, cancer, drug/alcohol addiction, or pre-gestational diabetes. Three women withdrew from the study, two miscarried after the time of enrollment, and one was lost to follow up, leaving 56 women followed-through delivery of a singleton infant. All women provided written, informed consent prior to engaging in study activities and all protocols were approved by the WIHRI institutional review board. The involvement of the Centers for Disease Control and Prevention (CDC) laboratory did not constitute engagement in human subjects research.

### Urine sample collection and quantification of urinary OPE metabolite concentrations

Urine sample collection and analysis was described previously (Romano et al. [21]). Briefly, we collected spot urine samples in polypropylene specimen cups during clinic visits at three time points during pregnancy: enrollment (12 ± 2 gestational weeks) and at two routine antenatal screening visits, the first for gestational diabetes screening (28 ± 2 gestational weeks) and the second for group B streptococcus screening (35 ± 1 gestational weeks). All 56 women provided at least one urine sample during pregnancy, 53 (95%) provided at least two samples, and 40 (71%) provided all three samples. Urine samples were immediately refrigerated following collection. Urine samples were vortexed for 30 s and specific gravity (SG) was measured using a handheld digital refractometer (ATAGO, PAS-10S) to quantify urine dilution. Urine was aliquoted into polypropylene cryovials and stored at − 80 °C within 24 h after collection. To provide an estimate of OPE exposure throughout pregnancy, we created a pooled urine sample for each woman using 1 mL of urine from each of her individual samples at the time of preparing the samples for shipment to the Division of Laboratory Sciences, National Center for Environmental Health, CDC (Atlanta, Georgia, USA). We shipped all samples on dry ice to the CDC, where they were stored at or below -20 °C until analysis.

Urinary metabolites of OPEs measured in women enrolled in this study showed good reproducibility throughout pregnancy (intraclass correlation coefficient (95% confidence interval): BCEP = 0.50 (0.37, 0.58); BDCPP = 0.60 (0.54, 0.66); and DPHP = 0.43; (0.36, 0.50) [21];). Therefore, we used OPE metabolite concentrations in pooled urine samples collected throughout pregnancy at approximately 12, 28 and 35 weeks gestation to reflect exposures occurring during the window of gestation. Three OPE metabolites (bis-2-chloroethyl phosphate [BCEP], bis (1,3-dichloro-2-propyl) phosphate [BDCPP], and DPHP) were detected in at least 74% of urine samples [21]. These are urinary metabolites of three of the most common OPEs detected in environmental media: tri-2-chloroethyl phosphate [TCEP], tri (1,3-dichloro-2-propyl) phosphate [TDCPP], and TPHP, respectively, though other OPEs, including 2-ethylhexyl diphenyl phosphate (EHDPHP), may also be precursors of some of these metabolites (e.g., DPHP). Analytical methods have been described in detail previously [39]. Briefly, the method uses an enzymatic hydrolysis of urinary conjugates followed by automated off-line solid phase extraction with a polymeric weak anion exchange cartridge to pre-concentrate the target compounds while minimizing potential urine matrix interferences. The deconjugated target analytes in the urine extract are separated on an ultra-high-performance liquid chromatography system with reversed phase chromatography and quantified by isotope dilution-negative ion electrospray ionization tandem mass spectrometry. Standard quality assurance and quality control measures used during analysis were within acceptable laboratory limits. Limits of detection (LODs) for the individual OPE metabolites ranged from 0.08 to 0.16 μg/L, depending on the analyte (Supplemental Material, Table S1). A value of LOD/√2 was assigned when OPE concentrations were below the LOD in pooled urine samples [40].

Concentrations of urinary metabolites of interest were SG-standardized using a modification of a previously described formula: *P*_*c*_ = *P* [*SG*_*ref*_-1/*SG*-1] [41], where *P*_*c*_ is the SG-standardized urinary metabolite concentration (μg/L), *P* is the concentration of the metabolite quantified in the urine sample (μg/L), *SG*_*ref*_ was the mean SG for all samples (1.017) and *SG* was the average SG across samples contributing to each individual’s pooled sample. Unstandardized OPE metabolite concentrations in individual and pooled urine samples were previously reported in Romano et al. [21]. Concentrations of urinary OPE metabolites were then log_2_-transformed to decrease the influence of extreme values throughout subsequent statistical analyses.

### Covariate information

At enrollment, women completed a brief questionnaire describing their highest level of education attained and household income. We abstracted additional demographic (maternal age and race), anthropometric (pre-pregnancy weight and height), and perinatal factors (parity) from the women’s medical records following delivery. Pre-pregnancy weight was available in the medical records of most women (81%). We substituted weight from the earliest prenatal care visit for nine women missing pre-pregnancy weight (average: 11 weeks gestation; range: 8–15 weeks gestation) when calculating body mass index (BMI, kg/m^2^) because weight gain in early pregnancy is generally not substantial [42].

### Gestational weight gain (GWG)

Last weight in pregnancy was abstracted from the maternal medical record (mean ± SD = 38.2 ± 2.3 gestational weeks) and GWG was calculated as last weight in pregnancy (kg) - pre-pregnancy weight (kg). Based on their pre-pregnancy BMI and calculated GWG, women were categorized as having limited, adequate, or excessive GWG based on the Institute of Medicine’s guidelines [43].

### Newborn anthropometry and feeding behaviors

Trained research staff obtained anthropometric measures of size (weight, length, and head and abdominal circumference) and body composition (suprailiac, thigh, tricep, and subscapular skinfold thickness) after birth (mean ± SD = 1.4 ± 1.2 days, range = 0–7) for 49 newborns delivered at WIHRI. Newborns delivered at a non-study hospital (*n* = 4) were not measured after birth, and 3 newborns were discharged prior to being measured. Staff measured newborn weight (Doran DS4100 Baby Scale), birth length (SECA 417 Length Board), and head and abdominal circumference (Guilick II Tape Measure, Model 67,020). They also measured skinfold thickness using Harpenden skinfold calipers. Each measurement was taken twice, and if the difference between the two measurements was outside of a pre-specified range (≥10 g for weight, ≥0.5 cm for length, head and abdominal circumference, and ≥ 0.5 mm for skinfold thickness), then a third measurement was recorded. We used the average of the two or three individual measurements for all analyses. Staff repeated all anthropometric measurements at approximately 6 weeks postpartum (mean ± SD = 6.8 ± 1.1, range = 4.4–9.1) during a study visit (*n* = 45).

At 6 weeks postpartum, mothers completed the Baby Eating Behavior Questionnaire (BEBQ) to describe infant feeding habits (*n* = 49) related to five appetitive traits in both bottle- and breastfed infants: general appetite, enjoyment of food, food responsiveness, slowness in eating, and satiety responsiveness [44]. The BEBQ was developed to evaluate infant feeding behavior that may infer susceptibility to weight gain later in life [44].

### Statistical analysis

We performed all statistical analyses using R version 3.6.1 and SAS software version 9.4 [45, 46]. We calculated descriptive statistics (e.g., mean, standard deviation (SD), range) for maternal and infant characteristics. We also calculated the overall median and interquartile range (IQR) of urinary OPE metabolite concentrations for all women in the study, as well as by GWG category. We used multiple linear regression to examine the influence of log_2_-transformed maternal urinary OPE metabolite concentrations on continuous GWG, controlling for covariates selected a priori based on their associations with the exposure and/or outcome in this study: maternal age at delivery, income, pre-pregnancy BMI, parity, and infant sex [21]. We evaluated associations between prenatal OPE exposure and infant gestational age at delivery and newborn size (weight, length, and head and abdominal circumference) using log_2_-transformed maternal urinary OPE metabolite concentrations and multiple linear regression, controlling for the same covariates as were used for GWG. Growth trajectories and body composition during early life have implications for obesity and cardiometabolic health outcomes across the life course [47–52]. We calculated the weekly rate of growth between birth and 6 weeks for each of four anthropometric measures of size (weight, length, head and abdominal circumference) and four measures of body composition (iliac, subscapular, tricep, and thigh skinfold thickness) as $\left(\frac{measuremet_{6- weeks}-{measurement}_{delivery}}{age\ {in\ days}_{6- weeks}- age\ {in\ days}_{delivery}}x7\right)$ and used multivariable linear regression adjusted for the same covariates as above to estimate the association of maternal log2-transformed OPE metabolite concentrations on rate of change in anthropometric measurements during the first 6 weeks. We also adjusted for birth weight in all weekly growth rate models for all anthropometric measures except for weight. Additionally, we evaluated associations between log_2_-transformed OPE metabolite concentrations and overall anthropometry for each of the size and body composition measurements during the first 6 weeks of life using linear mixed effects models with unstructured covariance to account for repeated anthropometric measurements at birth and 6 weeks postpartum for an individual. We investigated the inclusion of random intercepts in these models, but did not retain these terms based on model fit (Akaike Information Criteria). Collectively, these measures of overall body size and composition provide insight into subcutaneous adipose tissue distribution [53–55]. We adjusted linear mixed effects models for the same covariates as multiple linear regression models, as well as timing of six-week postpartum infant anthropometric measurements. Because existing literature shows effect modification of OPE exposure on birth outcomes by infant sex [19], we evaluated sex-specific effects by including an interaction (exposure*sex) term in multiple linear regression and linear mixed effects models for all OPE metabolites, for each birth outcome. For all regression modeling techniques, we used a complete case analysis approach due to minimal missing covariate information. Based on our evaluation of unadjusted associations between OPE metabolite concentrations and infant anthropometric measures, we do not believe model overfitting was a concern in our study despite the pilot-scale sample size and suite of covariates which were previously identified to predict OPE metabolite concentration in this cohort [21]. Finally, we estimated BEBQ subscales for infant feeding behavior corresponding to five appetitive traits: enjoyment of food, food responsiveness, slowness in eating, satiety responsiveness, and general appetite [44]. We then used log_2_-transformed maternal urinary OPE metabolite concentrations and multiple linear regression to evaluate the potential for OPE exposure to affect infant feeding behavior. These models were also adjusted for maternal age at delivery, income, pre-pregnancy BMI, parity, postpartum week of BEBQ response, and infant sex.

## Results

The average age of women (*n* = 56) in the study at the time of birth was 29.5 years old, with an average pre-pregnancy BMI of 27.9 mg/kg^2^ (Table 1). Of these women, 34 (64%) identified as non-Hispanic white, 25 (45%) had a minimum of a bachelor’s or other technical degree, and 22 (41%) were nulliparous (Table 1). Of the infants born to women enrolled in the study, 24 (44%) were female and 30 (56%) were male (Table 1). The mean (SD; range) gestational age of infants at delivery was 39.2 weeks (1.4; 35.9–41.3 weeks) and mean infant weight at birth was 3.1 kg (0.5; 1.7–4.2 kg) (Table 1). Median concentrations of OPE metabolites in pooled urine samples were 0.31 (IQR: 0.17, 0.54) μg/L for BCEP, 1.18 (IQR: 0.65, 2.20) μg/L for BDCPP, and 0.93 (IQR: 0.73, 1.95) μg/L for DPHP (Table 2).

**Table 1:** Descriptive statistics for maternal and infant characteristics (*n* = 56 maternal-infant pairs)

Cohort Characteristics	Mean ± SD	N Missing
	N (%)	
Maternal Age	29.5 ± 4.5	4
Maternal BMI (kg/m^2^)	27.9 ± 7.1	0
Non-Hispanic White	35 (64)	1
Maternal Education		0
High School or Less	15 (27)	–
Tech school/Some College	16 (29)	–
Bachelor’s/Graduate/Professional	25 (45)	–
Household Income		0
< $25,000	20 (36)	–
$25,000-100,000	18 (32)	–
> $100,000	18 (32)	–
Parous	32 (59)	2
Gestational week of delivery	39.2 ± 1.4	0
Infant sex: Female	24 (44)	2
Birth weight (kg)	3.1 ± 0.5	7
Infant weight at postpartum visit (kg)	4.9 ± 0.6	11
Week of postpartum visit	6.8 ± 1.1	11

**Table 2:** OPE metabolite concentration (μg/L) overall and by GWG category in pooled maternal urine samples

GWG Categories	N	OPE: Median (IQR)		
		BECP	BDCPP	DPHP
**Overall**	**56**	0.31 (0.17, 0.54)	1.18 (0.65, 2.20)	0.93 (0.73, 1.95)
**Limited**	**38**	0.35 (0.17, 0.60)	0.97 (0.59, 2.05)	0.87 (0.61, 1.66)
**Appropriate**	**17**	0.30 (0.19, 0.42)	1.84 (0.97, 5.05)	1.27 (0.87, 2.13)

Gestational weight gain categories were calculated according to the Institute of Medicine’s guidelines [43]. Only one woman in our study had excessive GWG

Concentrations of BCEP were similar between women with limited versus appropriate GWG, whereas concentrations of both BDCPP and DPHP were higher in women with appropriate GWG compared to those with limited GWG (Table 2). Only one woman in our study had excessive GWG. Maternal urinary OPE metabolites were weakly associated with increased GWG (Table S2). We did not observe strong associations between OPE metabolite concentrations and gestational age at delivery, infant size at birth, or evidence of effect modification of the relationship between OPE metabolite concentration and birth outcomes by infant sex (all exposure*sex *p*-values ≥0.29). Effect estimates among all infants are reported in Table 3.

**Table 3:** Associations between maternal urinary OPE metabolites and gestational age at delivery and infant anthropometry at birth

Outcome	log_**2**_(BCEP)	log_**2**_(BDCPP)	log_**2**_(DPHP)
	Beta (95% CI)	Beta (95% CI)	Beta (95% CI)
**Gestational Age at Delivery**	0.04 (−0.2, 0.29)	0.27 (−0.06, 0.6)	0.28 (−0.1, 0.65)
**Birth Weight**	0.09 (−0.01, 0.18)	0.08 (− 0.05, 0.21)	0.04 (− 0.12, 0.19)
**Birth Length**	− 0.02 (− 0.55, 0.5)	0.64 (− 0.03, 1.31)	0.17 (− 0.64, 0.98)
**Head Circumference**	−0.12 (− 0.55, 0.31)	0.09 (− 0.49, 0.66)	0.07 (− 0.6, 0.74)
**Abdominal Circumference**	0.03 (− 0.39, 0.44)	0.51 (− 0.02, 1.04)	−0.59 (−1.2, 0.03)

Anthropometric measurements were made at birth (mean ± SD = 1.4 ± 1.2 days old). OPE metabolite concentrations were log_2_-transformed and multiple linear regression models were adjusted for maternal age at delivery, income, pre-pregnancy BMI, parity and infant sex. Overall effect estimates are presented since all exposure*sex interaction terms were non-significant. Gestational age at delivery is reported in weeks; Anthropometric measures include: weight (kg), length (cm), head circumference (cm), and abdominal circumference (cm)

**Fig. 1:**
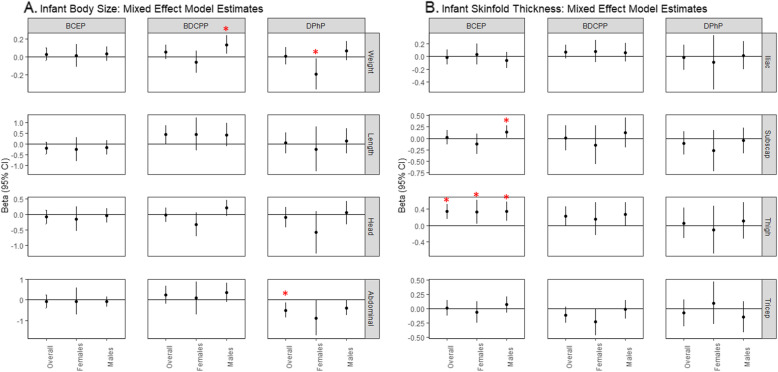
**a**-**b.** Linear mixed effects model results for infant size (**a**) and body composition (**b**) during the first 6 weeks of life: All infants and stratified by infant sex. Measurements of infant size and skinfold thickness were repeated at birth (mean ± SD = 1.4 ± 1.2 days) and approximately 6 weeks postpartum (mean ± SD = 6.8 ± 1.1 weeks). OPE metabolite concentrations were log_2_-transformed and linear mixed effects models were adjusted for maternal age at delivery, income, pre-pregnancy BMI, parity, infant sex and age at the time of measurement. Overall (female and male infants combined) and sex-specific effect estimates are presented with corresponding 95% confidence intervals. A red asterisk (*) denotes α < 0.05, indicating statistical significance. Anthropometric measures include: weight (kg), length (cm), head circumference (cm), abdominal circumference (cm) and skinfold thicknesses (mm). Effect estimates and 95% confidence intervals are reported in Table S4

Our assessment of weekly growth rate for infant anthropometric measures during the first 6 weeks of life showed that DPHP was positively associated with weekly growth in iliac skinfold thickness among all infants (β_overall_ = 0.10 mm, 95% CI_overall_: 0.02, 0.19; Table S3) and males (β_male_ = 0.12 mm, 95% CI_male_: 0.02, 0.23; Table S3; p-for-EM = 0.47), as well as weekly growth in tricep skinfold thickness among males (β_male_ = 0.10 mm, 95% CI_male_: 0.02, 0.19; Table S3; p-for-EM = 0.06). We did not observe associations between other OPE metabolites and other measures of weekly infant growth. We observed associations between OPE metabolites and overall body size and composition during the first 6 weeks of life. Overall, BDCPP was positively associated with measures of infant size, BCEP was positively associated with measures of infant body composition, and DPHP was negatively associated with infant size. Specifically, a doubling in BDCPP concentration in maternal urine was associated with a 0.44 cm longer infant length (95% CI_overall_: 0.01, 0.87**)** and 0.14 kg greater weight in males (95% CI_males_: 0.03, 0.24; Fig. 1a, Table S4; p-for-EM = 0.02). BCEP was positively associated with thigh skinfold thickness in all infants (β_overall_ = 0.34 mm, 95% CI_overall_: 0.16, 0.52) and with subscapular skinfold thickness in males only (β_males_ = 0.14 mm, 95% CI_males_: 0.002, 0.28; Figure 1b, Table S4; p-for-EM = 0.05). Greater maternal urinary DPHP was associated with smaller abdominal circumference (β_overall_ = − 0.50 cm, 95% CI_overall_: − 0.86, − 0.14) and weight among females (β_females_ = − 0.19 kg, 95% CI_females_: − 0.36, − 0.02; Fig. 1a, Table S4). However, DPHP was positively associated with weight in males (β_males_ = 0.07 kg, 95% CI_males_: − 0.04, 0.18; Fig. 1a, Table S4; p-for-EM = 0.02).

**Fig. 2:**
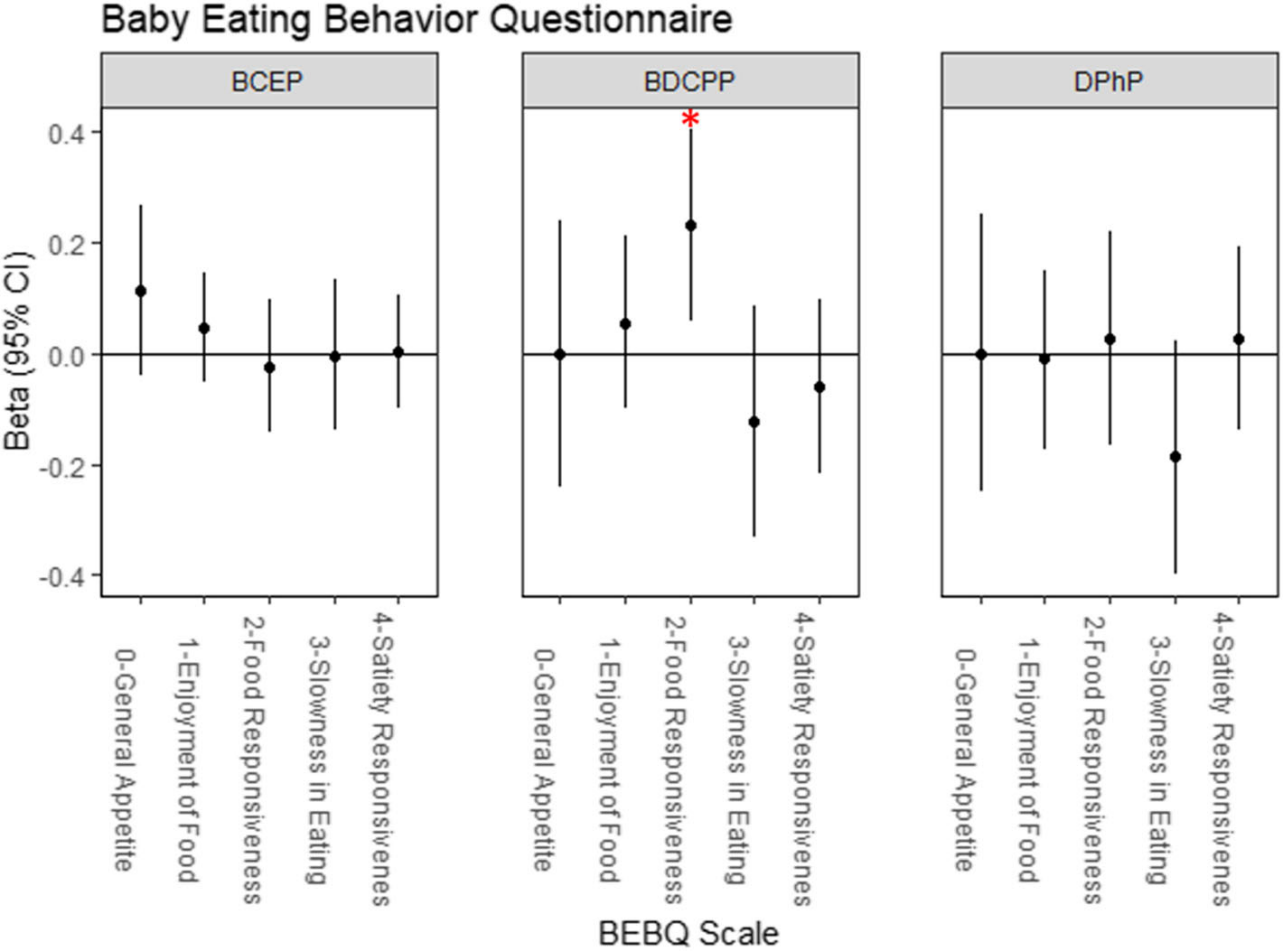
Multiple linear regression analyses for Baby Eating Behavior Questionnaire appetitive traits at 6 weeks postpartum. The BEBQ [44] was completed by mothers at approximately 6 weeks (mean ± SD = 6.8 ± 1.1 weeks) postpartum and mean trait-specific scores were calculated for four appetitive traits with multiple relevant questions on the BEBQ: enjoyment of food, food responsiveness, slowness in eating, satiety responsiveness. General appetite was assessed by a single question about overall infant appetite. OPE metabolite concentrations were log_2_-transformed and multiple linear regression models were adjusted for maternal age at delivery, income, pre-pregnancy BMI, parity, infant sex, and postpartum week of survey completion. A red asterisk (*) denotes α < 0.05, indicating statistical significance. Effect estimates and 95% confidence intervals are reported in Table S5

Among the five BEBQ appetitive traits, BDCPP was associated with increased food responsiveness (β = 0.23 points, 95% CI = 0.06, 0.40) (Fig. 2, Table S5) and DPHP was weakly associated with faster feeding speeds. BDCPP and DPHP did not strongly influence any other traits, and BCEP did not impact infant feeding behavior for any trait.

## Discussion

Toxicological studies suggest that OPEs are endocrine-disrupting compounds, and limited epidemiologic evidence suggests that exposure to these compounds during pregnancy may alter perinatal health outcomes. To extend the epidemiologic literature, we evaluated the associations of three urinary OPE metabolites (BCEP, BDCPP, DPHP) measured throughout pregnancy with GWG, gestational age at delivery, anthropometry, and feeding behavior. Exposure to OPEs during pregnancy was weakly associated with increased GWG. Our findings suggest that OPE exposure may affect infant growth, body size and composition, and feeding behavior during the first 6 weeks of life. BDCPP was positively associated with overall measures of infant size and infant responsiveness to food during the first 6 weeks postpartum. DPHP was negatively associated with overall infant size, but positively associated with weekly growth in skinfold thickness. BCEP was positively associated with measures of infant body composition. Collectively, these findings support previous studies suggesting that prenatal exposures to select OPEs may be compound- and sex-specific in their effects on infants.

Prior epidemiologic studies have reported positive associations between maternal urinary OPE metabolite concentrations and maternal weight and BMI [14, 21, 56]. In the present study, we further evaluated OPE metabolite concentrations as a predictor of GWG. The weak, positive relationship between urinary OPE metabolites and GWG that we identified adds a new, complimentary dimension to the existing narrative surrounding OPE exposure and maternal body size. One possible explanation for the association is that OPEs cause weight gain, possibly via perturbations to nuclear receptor signaling, leading to effects on sex and thyroid hormones, and lipid and glucose homeostasis [27–30, 32–34, 57]. Such modes of action were postulated as an explanation for associations observed between OPE exposure and pre-pregnancy BMI among pregnant women enrolled in a North Carolina-based cohort [56]. Healthy GWG is important for maternal and infant health during pregnancy and beyond [43, 58, 59]. Pregnancy represents a period of marked, complex changes to endocrine and metabolic physiology in women, including thyroid function and thyroid hormone levels, adipose tissue mass, function, and adipokine signaling, and endocrine functions of the placenta [43]. Because OPEs have been shown to act on similar endocrine signaling endpoints, which can affect body weight regulation, the period of pregnancy may represent a vulnerable period of exposure for OPE modulation of body weight. However, it is possible that increased body size may lead to increased OPE exposure via larger surface area for dermal absorption of OPEs or dietary patterns [21, 60–62]. Therefore, further research is needed to determine causality, as well as to confirm the suggestive positive relationship between OPE exposure and GWG in a larger study population.

Infant size and growth have been associated with the infant’s risk of obesity [47–51], with both small and large birth size increasing risk of adverse cardiometabolic health later in life [47–49, 63–67]. Even small changes in size at birth may have health consequences across the life course. While urinary OPE metabolite concentrations were not associated with infant anthropometrics at birth in this study, our findings suggest that OPE exposure during pregnancy may affect overall size and body composition and growth during early life. Anthropometric measurements at 6 weeks postpartum suggested OPE- and sex-specific impacts on overall infant size and body composition during early life. BDCPP was positively associated with weight in males, and length in all infants. Conversely, DPHP was negatively associated with anthropometry, including reduced weight in female infants and smaller abdominal circumference for all infants. DPHP was positively associated with weekly increases in skinfold thicknesses. These findings suggest that DPHP may encourage “catch up” growth and subcutaneous fat accretion during early life, which is a known risk factor for chronic diseases across the life course [67, 68]. Taken together, our study lends further support to existing literature showing compound- and sex-specific effects of OPE exposure on perinatal growth and development, notably extending these findings beyond prenatal development, to the early neonatal developmental period [19]. While this pilot study was under powered to evaluate effects of OPE metabolites mixtures on perinatal health, additional epidemiologic and toxicological studies are needed to better contextualize our findings in relation to environmentally-relevant mixtures. Additionally, Hoffman et al. [19] found that OPE exposure was associated with preterm birth in female infants, though we did not observe associations between maternal urinary OPE metabolites with length of gestation in our study.

Anthropometric measurements offer an inexpensive and informative means of assessing size and body composition across the life course and can serve as an important research tool for studying effects of endocrine-disrupting compounds on growth and development. Infant anthropometry is a particularly important perinatal clinical and research tool since other methods (e.g., magnetic resonance imaging, bioelectrical impedance analysis) of evaluating body composition in children and adults are impractical for infants [69]. Measures such as weight, length, and head and abdominal circumference can provide useful information about infant size and may be indicators of infant overall growth and development. Skinfold thickness measurements correlate well with subcutaneous adipose distribution (e.g., peripheral versus truncal) and inform estimates of relative fat mass (FM) and fat free mass (FFM) (reviewed in Demerath and Fields [53]). Yet, there is debate about the reliability of neonatal anthropometry, particularly skinfold thicknesses because measurement error may arise from inter-measurer variability [70]. Although maternal urinary OPE metabolites were generally not associated with measures of body composition, DPHP was associated with higher weekly growth rates for iliac and tricep skinfold thickness during the first 6 weeks postpartum and BCEP was associated with higher overall subscapular skinfold thickness in males and thigh skinfold thickness in all infants in our study. Due to its pilot nature, our study does not include record of measurer, however, all staff received the same training for collecting anthropometric measurements. Although measurer is unlikely to be associated with exposure, future studies should consider adjustment for measurer into statistical analyses, particularly for measures of body composition. Collectively, our findings indicate that future research is needed to assess the influence of OPEs on body composition in early life.

We also used the BEBQ to evaluate whether gestational OPE exposure may affect infant feeding behavior as a possible explanation for changes in anthropometry. BDCPP was associated with greater food responsiveness, which has been identified as a risk factor for rapid infant weight gain in previous studies using the BEBQ [44, 71, 72]. Notably, the positive association between BDCPP concentration and food responsiveness corresponds with the positive association we observed between prenatal BDCPP concentrations and infant weight gain, especially in males. BCEP was suggestively associated with increased general appetite, which also indicates susceptibility for increased weight gain [44]. These findings may suggest that increases in infant size and subcutaneous fat accretion may be associated with increased appetite and possibly higher caloric intake. The negative association between DPHP and “Slowness in Eating” suggests that DPHP may increase feeding rate in infants and may explain the faster rates of subcutaneous fat accretion we observed [72] even though DPHP was negatively associated with overall measures of infant size (e.g., weight and abdominal circumference) in our cohort. Collectively, these findings underscore the need to better understand the capacity for prenatal exposure to select OPEs to affect infant feeding behavior immediately postpartum, and to further investigate connections between infant feeding behavior and anthropometry in the context of prenatal OPE exposure.

Our study has several strengths and limitations. Importantly, our study (1) used multiple prospective urine samples from pregnancy to characterize OPE exposure, (2) evaluated prenatal OPE exposure in the context of infant growth during early life using repeated anthropometric measures at birth and 6 weeks, and (3) analyzed the effects of prenatal OPE exposure on infant feeding behavior. The prospective study designs afforded temporal clarity of OPE exposure with birth and infant outcomes. The quantification of OPE metabolites in pooled urine samples provides an estimate of OPE exposure throughout pregnancy and reduces the potential influence of temporal variability, which has been shown in prior studies [56, 73]. Another strength is that study participants were enrolled at WIHRI, which provides care for the majority (80%) of deliveries in Rhode Island, allowing us to recruit from a population that represents the general Rhode Island population.

Nevertheless, we recognize several key limitations, including the pilot-scale sample size and the potential for measurement error and unmeasured confounding. The pilot-scale sample size presents challenges in terms of statistical power and primarily allows us to generate hypotheses for future study. Despite the limitation of working with a small sample of maternal-child pairs, like Hoffman et al. [19], our findings show evidence of effect modification of the association between OPE exposure and infant anthropometry by sex, which is encouraging. A larger study may want to consider using the augmented cross-product term approach to evaluate effect modification in order to account for potentially different confounding structures by infant sex [74], however we did not have sufficient statistical power in our pilot study to do so. Thus, our results of effect modification of the association between maternal urinary OPE metabolites and infant anthropometry are exploratory. It is also important to acknowledge the potential for exposure misclassification based on OPE metabolite measurements, particularly for women who provided only one urine sample, because of possible seasonal variability in urinary concentrations of OPE metabolites, and of measurement error in infant anthropometry. Nevertheless, our data collection methods (quantifying OPE metabolites in pooled urine samples collected throughout pregnancy; using the average of two to three measurements for each anthropometric outcome) are proven techniques to minimize such potential limitations (e.g., [21, 75, 76]). As with all environmental epidemiology studies, residual confounding by unknown or unmeasured co-exposures that influence the outcomes of interest may be present.

## Conclusions

Our findings suggest that OPE exposure during gestation can affect infant growth. BDCPP was associated with both increased infant growth and increased food responsiveness in the first 6 weeks of life. DPHP was positively associated with rates of subcutaneous fat accretion, but inversely associated with measures of infant size. BCEP was positively associated with measures of infant body composition. Compound- and sex-specific patterns of association between OPE metabolites and measures of growth observed here and shown previously, suggest that OPEs may act through different mechanisms of action. Since birth size, growth trajectories, and feeding behavior during early life have implications for obesity and cardiometabolic health outcomes, the ability for OPEs to interfere with these processes may have developmental and metabolic consequences across the life course. Further, our study identified weak positive associations between OPE exposure during pregnancy and GWG, which also can impact maternal and infant cardiometabolic health across the life course. Given the pilot-scale of this study, we recommend these findings be followed up in a large cohort. Further research is also needed to understand the mechanistic basis for endocrine action of OPEs, particularly relating to cardiometabolic health and feeding behavior. Evaluating associations between maternal urinary OPE metabolites at specific time points throughout pregnancy in future research may provide useful information about susceptible windows of exposure and/or modes of OPE action on perinatal health outcomes.

## Supplementary information

**Additional file 1: Table S1.** Parent OPE, limits of detection and detection frequency of associated urinary metabolites. **Table S2.** Maternal urinary OPE metabolite concentration was weakly associated with increased GWG. **Table S3.** Associations between urinary OPE metabolite concentration and weekly change in infant anthropometric measurement between birth and six weeks postpartum. **Table S4.** Linear mixed effects models for repeated measurements of infant anthropometrics at birth and six weeks postpartum. **Table S5.** Baby Eating Behavior Questionnaire (BEBQ) assessment of infant feeding behavior.

## Data Availability

Investigators interested in accessing datasets analyzed in the current study should contact Drs Kathryn Crawford (kcrawford@middlebury.edu) and Megan Romano (Megan.E.Romano@dartmouth.edu) and provide a description of their proposed project. Requests will be reviewed primarily to ensure that they do not overlap with extant projects, and datasets will be made available upon reasonable request.

## Abbreviations

BCEP: bis-2-chloroethyl phosphate
BDCPP: bis (1,3-dichloro-2-propyl) phosphate
BEBQ: Baby Eating Behavior Questionnaire
BMI: body mass index
DPHP: diphenyl phosphate
GWG: gestational weight gain
LME: linear mixed effects
LOD: limit of detection
OPE: Organophosphate ester
SG: specific gravity

## References

[1] Sjodin A, Jones R, Wong L, Caudill S, Calafat A (2019). Polybrominated Diphenyl ethers and biphenyl in serum: time trend study from the National Health and nutrition examination survey for years 2005/06 through 2013/14. Environ Sci Technol.

[2] Hoffman K, Butt CM, Webster TF, Preston EV, Hammel SC, Makey C (2017). Temporal trends in exposure to organophosphate flame retardants in the United States. Environ Sci Technol Lett.

[3] Stapleton HM, Sharma S, Getzinger G, Ferguson PL, Gabriel M, Webster TF (2012). Novel and high volume use flame retardants in US couches reflective of the 2005 PentaBDE Phase Out. Environ Sci Technol.

[4] Cooper EM, Kroeger G, Davis K, Clark CR, Ferguson PL, Stapleton HM (2016). Results from screening polyurethane foam based consumer products for flame retardant chemicals: assessing impacts on the change in the furniture flammability standards. Environ Sci Technol.

[5] Doherty BT, Hammel SC, Daniels JL, Stapleton HM, Hoffman K (2019). Organophosphate esters: are these flame retardants and plasticizers affecting Children’s health?. Curr Environ Heal Reports.

[6] van der Veen I, de Boer J (2012). Phosphorus flame retardants: properties, production, environmental occurrence, toxicity and analysis. Chemosphere.

[7] Stapleton HM, Klosterhaus S, Eagle S, Fuh J, Meeker JD, Blum A (2009). Detection of Organophosphate Flame Retardants in Furniture Foam and U.S. House Dust. Environ Sci Technol.

[8] Wei G-L, Li D-Q, Zhuo M-N, Liao Y-S, Xie Z-Y, Guo T-L (2015). Organophosphorus flame retardants and plasticizers: Sources, occurrence, toxicity and human exposure. Environ Pollut.

[9] Meeker JD, Stapleton HM (2009). House dust concentrations of organophosphate flame retardants in relation to hormone levels and semen quality parameters. Environ Health Perspect.

[10] Xu F, Giovanoulis G, van Waes S, Padilla-Sanchez JA, Papadopoulou E, Magnér J (2016). Comprehensive study of human external exposure to organophosphate flame retardants via air, dust, and hand wipes: the importance of sampling and assessment strategy. Environ Sci Technol.

[11] Dodson RE, Perovich LJ, Covaci A, Van den Eede N, Ionas AC, Dirtu AC (2012). After the PBDE Phase-Out: a broad suite of flame retardants in repeat house dust samples from California. Environ Sci Technol.

[12] Hoffman K, Garantziotis S, Birnbaum LS, Stapleton HM. Monitoring indoor exposure to organophosphate flame retardants: hand wipes and house dust. Environ Health Perspect. 2014; Available from: http://ehp.niehs.nih.gov/1408669. Cited 2018 Sep 4. 10.1289/ehp.1408669 PMC4314253 25343780

[13] Mendelsohn E, Hagopian A, Hoffman K, Butt CM, Lorenzo A, Congleton J (2016). Nail polish as a source of exposure to triphenyl phosphate. Environ Int.

[14] Carignan CC, Mínguez-Alarcón L, Butt CM, Williams PL, Meeker JD, Stapleton HM, et al. Urinary concentrations of organophosphate flame retardant metabolites and pregnancy outcomes among women undergoing in vitro fertilization. Environ Health Perspect. 2017;125(8). Available from: http://ehp.niehs.nih.gov/EHP1021. Cited 2018 Sep 4. 10.1289/EHP1021 PMC5783651 28858831

[15] Carignan CC, McClean MD, Cooper EM, Watkins DJ, Fraser AJ, Heiger-Bernays W (2013). Predictors of tris (1,3-dichloro-2-propyl) phosphate metabolite in the urine of office workers. Environ Int.

[16] Hoffman K, Fang M, Horman B, Patisaul HB, Garantziotis S, Birnbaum LS, et al. Urinary Tetrabromobenzoic Acid (TBBA) as a biomarker of exposure to the flame retardant mixture firemaster® 550. Environ Health Perspect. 2014; Available from: http://ehp.niehs.nih.gov/1308028. Cited 2018 Sep 4. 10.1289/ehp.1308028 PMC4154220 24823833

[17] Hoffman K, Butt CM, Chen A, Limkakeng AT, Stapleton HM (2015). High exposure to organophosphate flame retardants in infants: associations with baby products. Environ Sci Technol.

[18] Meeker JD, Cooper EM, Stapleton HM, Hauser R (2013). Urinary metabolites of organophosphate flame retardants: temporal variability and correlations with house dust concentrations. Environ Health Perspect.

[19] Hoffman K, Stapleton HM, Lorenzo A, Butt CM, Adair L, Herring AH (2018). Prenatal exposure to organophosphates and associations with birthweight and gestational length. Environ Int.

[20] Castorina R, Butt C, Stapleton HM, Avery D, Harley KG, Holland N, et al. Flame retardants and their metabolites in the homes and urine of pregnant women residing in California (the CHAMACOS cohort). Chemosphere. 2017; 179:159–66. Available from: https://www.sciencedirect.com/science/article/pii/S0045653517304472. Cited 2018 Sep 6. 10.1016/j.chemosphere.2017.03.076 PMC5491392 28365501

[21] Romano ME, Hawley NL, Eliot M, Calafat AM, Jayatilaka NK, Kelsey K (2017). Variability and predictors of urinary concentrations of organophosphate flame retardant metabolites among pregnant women in Rhode Island. Environ Heal.

[22] Ospina M, Jayatilaka NK, Wong L-Y, Restrepo P, Calafat AM (2018). Exposure to organophosphate flame retardant chemicals in the U.S. general population: data from the 2013-2014 National Health and nutrition examination survey. Environ Int.

[23] Fromme H, Lahrz T, Kraft M, Fembacher L, Mach C, Dietrich S (2014). Organophosphate flame retardants and plasticizers in the air and dust in German daycare centers and human biomonitoring in visiting children (LUPE 3). Environ Int.

[24] Van den Eede N, Heffernan AL, Aylward LL, Hobson P, Neels H, Mueller JF, et al. Age as a determinant of phosphate flame retardant exposure of the Australian population and identification of novel urinary PFR metabolites. Environ Int. 2015;74:1–8. Available from: https://www.sciencedirect.com/science/article/pii/S0160412014002724. Cited 2018 Sep 6. 10.1016/j.envint.2014.09.005 25277340

[25] Reemtsma T, Lingott J, Roegler S (2011). Determination of 14 monoalkyl phosphates, dialkyl phosphates and dialkyl thiophosphates by LC-MS/MS in human urinary samples. Sci Total Environ.

[26] Cequier E, Sakhi AK, Marcé RM, Becher G, Thomsen C (2015). Human exposure pathways to organophosphate triesters — A biomonitoring study of mother–child pairs. Environ Int.

[27] Zhang Q, Wang J, Zhu J, Liu J, Zhao M (2017). Potential glucocorticoid and mineralocorticoid effects of nine organophosphate flame retardants. Environ Sci Technol.

[28] Kojima H, Takeuchi S, Itoh T, Iida M, Kobayashi S, Yoshida T (2013). In vitro endocrine disruption potential of organophosphate flame retardants via human nuclear receptors. Toxicology.

[29] Belcher SM, Cookman CJ, Patisaul HB, Stapleton HM (2014). In vitro assessment of human nuclear hormone receptor activity and cytotoxicity of the flame retardant mixture FM 550 and its triarylphosphate and brominated components. Toxicol Lett.

[30] Suzuki G, Tue NM, Malarvannan G, Sudaryanto A, Takahashi S, Tanabe S (2013). Similarities in the endocrine-disrupting potencies of indoor dust and flame retardants by using human osteosarcoma (u2os) cell-based reporter gene assays. Environ Sci Technol.

[31] Dishaw LV, Macaulay LJ, Roberts SC, Stapleton HM (2014). Exposures, mechanisms, and impacts of endocrine-active flame retardants. Curr Opin Pharmacol.

[32] Pillai HK, Fang M, Beglov D, Kozakov D, Vajda S, Stapleton HM (2014). Ligand binding and activation of PPARγ by Firemaster® 550: effects on adipogenesis and osteogenesis in vitro. Environ Health Perspect.

[33] Du Z, Zhang Y, Wang G, Peng J, Wang Z, Gao S (2016). TPhP exposure disturbs carbohydrate metabolism, lipid metabolism, and the DNA damage repair system in zebrafish liver. Sci Rep.

[34] Patisaul HB, Roberts SC, Mabrey N, McCaffrey KA, Gear RB, Braun J (2013). Accumulation and Endocrine Disrupting Effects of the Flame Retardant Mixture Firemaster ® 550 in Rats: An Exploratory Assessment. J Biochem Mol Toxicol.

[35] Wang D, Zhu W, Chen L, Yan J, Teng M, Zhou Z (2018). Neonatal triphenyl phosphate and its metabolite diphenyl phosphate exposure induce sex- and dose-dependent metabolic disruptions in adult mice. Environ Pollut.

[36] Preston EV, McClean MD, Claus Henn B, Stapleton HM, Braverman LE, Pearce EN (2017). Associations between urinary diphenyl phosphate and thyroid function. Environ Int.

[37] Meeker JD, Cooper EM, Stapleton HM, Hauser R (2013). Exploratory analysis of urinary metabolites of phosphorus-containing flame retardants in relation to markers of male reproductive health. Endocr Disruptors.

[38] Carignan CC, Mínguez-Alarcón L, Williams PL, Meeker JD, Stapleton HM, Butt CM, et al. Paternal urinary concentrations of organophosphate flame retardant metabolites, fertility measures, and pregnancy outcomes among couples undergoing in vitro fertilization. Environ Int. 2018; 111:232–8. Available from: http://www.ncbi.nlm.nih.gov/pubmed/29241080. Cited 2018 Sep 25. 10.1016/j.envint.2017.12.005 PMC5800983 29241080

[39] Jayatilaka NK, Restrepo P, Williams L, Ospina M, Valentin-Blasini L, Calafat AM (2017). Quantification of three chlorinated dialkyl phosphates, diphenyl phosphate, 2,3,4,5-tetrabromobenzoic acid, and four other organophosphates in human urine by solid phase extraction-high performance liquid chromatography-tandem mass spectrometry. Anal Bioanal Chem.

[40] Hornung RW, Reed LD (1990). Estimation of average concentration in the presence of nondetectable values. Appl Occup Environ Hyg.

[41] Duty SM, Ackerman RM, Calafat AM, Hauser R (2005). Personal care product use predicts urinary concentrations of some phthalate monoesters. Environ Health Perspect.

[42] Deierlein AL, Siega-Riz AM, Herring A (2008). Dietary energy density but not glycemic load is associated with gestational weight gain. Am J Clin Nutr.

[43] Rasmussen KM, Yaktine AL (2009). Composition and components of gestational weight Gain: physiology and metabolism. Weight Gain during pregnancy: reexamining the guidelines.

[44] Llewellyn CH, van Jaarsveld CHM, Johnson L, Carnell S, Wardle J (2011). Development and factor structure of the baby eating behaviour questionnaire in the gemini birth cohort. Appetite.

[45] R Core Team (2019). R: A language and environment for statistical computing.

[46] SAS Intitute Inc (2019). SAS software.

[47] Jones-Smith JC, Neufeld LM, Laraia B, Ramakrishnan U, Garcia-Guerra A, Fernald LCH (2013). Early life growth trajectories and future risk for overweight. Nutr Diabetes.

[48] Oken E, Gillman MW (2003). Fetal origins of obesity. Obes Res.

[49] Taveras EM, Rifas-Shiman SL, Belfort MB, Kleinman KP, Oken E, Gillman MW (2009). Weight status in the first 6 months of life and obesity at 3 years of age. Pediatrics.

[50] Singhal A, Lucas A (2004). Early origins of cardiovascular disease: is there a unifying hypothesis?. Lancet.

[51] Woo Baidal JA, Locks LM, Cheng ER, Blake-Lamb TL, Perkins ME, Taveras EM (2016). Risk factors for childhood obesity in the first 1,000 days: a systematic review. Am J Prev Med.

[52] Admassu B, Wells JCK, Girma T, Belachew T, Ritz C, Owino V (2018). Body composition during early infancy and its relation with body composition at 4 years of age in Jimma, an Ethiopian prospective cohort study. Nutr Diabetes.

[53] Demerath EW, Fields DA (2014). Body composition assessment in the infant. Am J Hum Biol.

[54] Lohman TG (1989). Assessment of body composition in children. Pediatr Exerc Sci.

[55] Gerver WJM, De Bruin R (1996). Body composition in children based on anthropometric data. A presentation of normal values. Eur J Pediatr.

[56] Hoffman K, Lorenzo A, Butt C, Adair L, Herring AH, Stapleton HM (2017). Predictors of urinary flame retardant concentration among pregnant women. Environ Int.

[57] Wang Q, Liang K, Liu J, Yang L, Guo Y, Liu C (2013). Exposure of zebrafish embryos/larvae to TDCPP alters concentrations of thyroid hormones and transcriptions of genes involved in the hypothalamic–pituitary–thyroid axis. Aquat Toxicol.

[58] Siega-Riz AM, Viswanathan M, Moos M-K, Deierlein A, Mumford S, Knaack J (2009). A systematic review of outcomes of maternal weight gain according to the Institute of Medicine recommendations: birthweight, fetal growth, and postpartum weight retention. Am J Obstet Gynecol.

[59] Diesel JC, Eckhardt CL, Day NL, Brooks MM, Arslanian SA, Bodnar LM (2015). Gestational Weight gain and offspring longitudinal growth in early life. Ann Nutr Metab.

[60] Kim H, Rebholz CM, Wong E, Buckley JP (2020). Urinary organophosphate ester concentrations in relation to ultra-processed food consumption in the general US population. Environ Res.

[61] Frederiksen M, Stapleton HM, Vorkamp K, Webster TF, Jensen NM, Sørensen JA (2018). Dermal uptake and percutaneous penetration of organophosphate esters in a human skin ex vivo model. Chemosphere..

[62] Li J, Zhao L, Letcher RJ, Zhang Y, Jian K, Zhang J (2019). A review on organophosphate Ester (OPE) flame retardants and plasticizers in foodstuffs: Levels, distribution, human dietary exposure, and future directions. Environ Int.

[63] Roseboom TJ, van der Meulen JH, Osmond C, Barker DJ, Ravelli AC, Schroeder-Tanka JM (2000). Coronary heart disease after prenatal exposure to the Dutch famine, 1944-45. Heart.

[64] Barker DJP (1995). Fetal origins of coronary heart disease. BMJ.

[65] Jones-Smith JC, Fernald LCH, Neufeld LM (2007). Birth size and accelerated growth during infancy are associated with increased odds of childhood overweight in mexican children. J Am Diet Assoc.

[66] Gluckman PD, Hanson MA, Beedle AS, Raubenheimer D (2008). Fetal and neonatal pathways to obesity. Obesity and metabolism.

[67] Baird J, Fisher D, Lucas P, Kleijnen J, Roberts H, Law C (2005). Being big or growing fast: systematic review of size and growth in infancy and later obesity. BMJ.

[68] Desai M, Beall M, Ross MG (2013). Developmental origins of obesity: programmed adipogenesis.

[69] Romano ME, Savitz DA, Braun JM (2014). Challenges and future directions to evaluating the association between prenatal exposure to endocrine-disrupting chemicals and childhood obesity. Curr Epidemiol Rep.

[70] Sopher A, Shen W, Pietrobelli A, Heymsfield SB, Lohman TG, Wang Z, Going SB (2005). Pediatric body composition methods. Human body composition.

[71] Patel N, Dalrymple KV, Briley AL, Pasupathy D, Seed PT, Flynn AC (2018). Mode of infant feeding, eating behaviour and anthropometry in infants at 6-months of age born to obese women – a secondary analysis of the UPBEAT trial. BMC Pregnancy Childbirth.

[72] Mallan KM, Daniels LA, de Jersey SJ (2014). Confirmatory factor analysis of the baby eating behaviour questionnaire and associations with infant weight, gender and feeding mode in an australian sample. Appetite.

[73] Cao Z, Xu F, Covaci A, Wu M, Yu G, Wang B (2014). Differences in the seasonal variation of brominated and phosphorus flame retardants in office dust. Environ Int.

[74] Buckley JP, Doherty BT, Keil AP, Engel SM (2017). Statistical approaches for estimating sex-specific effects in endocrine disruptors research. Environ Health Perspect.

[75] West J, Manchester B, Wright J, Lawlor DA, Waiblinger D (2011). Reliability of routine clinical measurements of neonatal circumferences and research measurements of neonatal skinfold thicknesses: findings from the Born in Bradford study. Paediatr Perinat Epidemiol.

[76] Vernet C, Philippat C, Agier L, Calafat AM, Ye X, Lyon-Caen S (2019). An empirical validation of the within-subject biospecimens pooling approach to minimize exposure misclassification in biomarker-based studies. Epidemiology..

