# Supplementary material for: Maternal urinary concentrations of organophosphate ester metabolites: associations with gestational weight gain, early life anthropometry, and infant eating behaviors among mothers-infant pairs in Rhode Island
Source: Environ Health. 2020 Sep 11;19:97. doi: 10.1186/s12940-020-00648-0 (PMC7488675; doi:10.1186/s12940-020-00648-0)
Supplement: Supplementary file 1 — Additional file 1: Table S1. Parent OPE, limits of detection and detection frequency of associated urinary metabolites. Table S2. Maternal urinary OPE metabolite concentration was weakly associated with increased GWG. Table S3. Associations between urinary OPE metabolite concentration and weekly change in infant anthropometric measurement between birth and six weeks postpartum. Table S4. Linear mixed effects models for repeated measurements of infant anthropometrics at birth and six weeks postpartum. Table S5. Baby Eating Behavior Questionnaire (BEBQ) assessment of infant feeding behavior. [file 12940_2020_648_MOESM1_ESM.docx]

**Supplemental Material**

**Maternal Urinary Concentrations of Organophosphate Ester Metabolites: Associations with Gestational Weight Gain, Early Life Anthropometry, and Infant Eating Behaviors among Mothers-Infant Pairs in Rhode Island**

Kathryn A. Crawford, Nicola Hawley, Antonia M. Calafat, Nayana K. Jayatilaka, Rosemary J. Froehlich, Phinnara Has, Lisa G. Gallagher, David A. Savitz, Joseph M. Braun, Erika F. Werner, Megan E. Romano

**Table of Contents**

Page 2: Table S1: Parent OPE, limits of detection and detection frequency of associated urinary metabolites

Page 3: Table S2: Maternal urinary OPE metabolite concentration was weakly associated with increased GWG.

Page 4: Table S3: Associations between urinary OPE metabolite concentration and weekly change in infant anthropometric measurement between birth and six weeks postpartum.

Page 5: Table S4: Linear mixed effects models for repeated measurements of infant anthropometrics at birth and six weeks postpartum.

Page 7: Table S5: Baby Eating Behavior Questionnaire (BEBQ) assessment of infant feeding behavior.

## SUPPLEMENTAL MATERIAL

**Table S1:** Parent OPE, limits of detection and detection frequency of associated urinary metabolites.

|  |  |  | Detection frequency (%) | | | | |
| --- | --- | --- | --- | --- | --- | --- | --- |
| Parent OPE | **Urinary Metabolite (Abbreviation)** | **LOD**  **(µg/L)** | **12 weeks** | **28**  **weeks** | **35**  **weeks** | **Pool** | **Overall ^a^** |
| Triphenyl phosphate | Diphenyl phosphate (DPHP) | 0.16 | 93 | 96 | 96 | 95 | 95 |
| Tris-(1,3-dichloro-2-propyl) phosphate | Bis-(1,3-dichloro-2-propyl) phosphate (BDCPP) | 0.11 | 86 | 94 | 98 | 97 | 93 |
| Tris-2-chloroethyl phosphate | Bis-2-chloroethyl phosphate (BCEP) | 0.08 | 66 | 73 | 71 | 83 | 74 |

^a^ Detection frequencies across all samples measured regardless of timing among all 59 women that provided at least one urine sample.

| **Table S2.** Maternal urinary OPE metabolite concentration was weakly associated with increased GWG. | | |
| --- | --- | --- |
| **log(BCEP)** | **log(BDCPP)** | **log(DPhP)** |
| **Beta (95% CI)** | **Beta (95% CI)** | **Beta (95% CI)** |
| 0.41 (-0.51, 1.32) | 0.75 (-0.51, 2.00) | 1.07 (-0.34, 2.48) |
| GWG (kg) was calculated as last weight in pregnancy (mean±SD=38.2±2.3 gestational weeks) minus pre-pregnancy weight. OPE metabolite concentrations were log_2_-transformed and multiple linear regression models were adjusted for maternal age at delivery, income, pre-pregnancy BMI, parity and infant sex. | | |

**Table S3.** Associations between urinary OPE metabolite concentration and weekly change in infant anthropometric measurement between birth and six weeks postpartum.

| **Anthropometric Measurement** | **Beta (95% CI)** | | |  |
| --- | --- | --- | --- | --- |
| **BCEP** | **Overall** | **Females** | **Males** | **p-for-EM** |
| Weight (kg/week) | -0.01 (-0.03, 0.01) | -0.03 (-0.05, 0.01) | -0.00 (-0.03, 0.02) | 0.34 |
| Length (cm/week) | -0.04 (-0.12, 0.04) | -0.09 (-0.22, 0.04) | -0.01 (-0.11, 0.09) | 0.35 |
| Abdominal circumference (cm/week) | -0.05 (-0.14, 0.04) | -0.08 (-0.23, 0.07) | -0.03 (-0.15, 0.09) | 0.61 |
| Head circumference (cm/week) | 0.01 (-0.05, 0.07) | 0.04 (-0.06, 0.14) | -0.00 (-0.08, 0.08) | 0.51 |
| Skinfold thickness (mm/week) |  |  |  |  |
| Iliac | 0.01 (-0.05, 0.07) | -0.02 (-0.11, 0.07) | 0.04 (-0.04, 0.12) | 0.30 |
| Thigh | -0.02 (-0.10, 0.06) | -0.03 (-0.16, 0.11) | -0.02 (-0.13, 0.09) | 0.93 |
| Tricep | -0.01 (-0.06, 0.04) | -0.02 (-0.10, 0.06) | -0.01 (-0.07, 0.06) | 0.80 |
| Subscapular | -0.00 (-0.06, 0.05) | -0.02 (-0.11, 0.06) | 0.01 (-0.06, 0.08) | 0.52 |
|  |  |  |  |  |
| BDCPP |  |  |  |  |
| Weight (kg/week) | 0.00 (-0.04, 0.03) | -0.01 (-0.06, 0.04) | 0.00 (-0.04, 0.04) | 0.66 |
| Length (cm/week) | -0.06 (-0.19, 0.07) | -0.03 (-0.22, 0.17) | -0.08 (-0.24, 0.09) | 0.69 |
| Abdominal circumference (cm/week) | -0.10 (-0.25, 0.05) | -0.10 (-0.32, 0.13) | -0.11 (-0.29, 0.08) | 0.94 |
| Head circumference (cm/week) | -0.02 (-0.11, 0.08) | -0.07 (-0.22, 0.08) | 0.02 (-0.10, 0.14) | 0.34 |
| Skinfold thickness (mm/week) |  |  |  |  |
| Iliac | 0.07 (-0.02, 0.16) | 0.05 (-0.09, 0.18) | 0.09 (-0.03, 0.21) | 0.62 |
| Thigh | 0.07 (-0.06, 0.20) | 0.10 (-0.10, 0.30) | 0.05 (-0.12, 0.22) | 0.70 |
| Tricep | -0.01 (-0.10, 0.07) | -0.05 (-0.18, 0.08) | 0.01 (-0.10, 0.12) | 0.48 |
| Subscapular | 0.02 (-0.06, 0.11) | 0.07 (-0.06, 0.20) | -0.01 (-0.12, 0.10) | 0.34 |
|  |  |  |  |  |
| DPHP |  |  |  |  |
| Weight (kg/week) | -0.01 (-0.04, 0.02) | -0.02 (-0.09, 0.04) | -0.01 (-0.04, 0.03) | 0.64 |
| Length (cm/week) | -0.06 (-0.18, 0.06) | -0.01 (-0.28, 0.25) | -0.07 (-0.22, 0.07) | 0.69 |
| Abdominal circumference (cm/week) | 0.05 (-0.09, 0.19) | 0.03 (-0.28, 0.33) | 0.06 (-0.11, 0.23) | 0.86 |
| Head circumference (cm/week) | -0.04 (-0.13, 0.05) | -0.16 (-0.35, 0.03) | -0.00 (-0.11, 0.10) | 0.16 |
| Skinfold thickness (mm/week) |  |  |  |  |
| Iliac | **0.10 (0.02, 0.19)** | 0.06 (-0.10, 0.22) | **0.12 (0.02, 0.23)** | 0.47 |
| Thigh | 0.06 (-0.06, 0.19) | -0.08 (-0.33, 0.18) | 0.11 (-0.04, 0.25) | 0.22 |
| Tricep | 0.06 (-0.01, 0.14) | -0.07 (-0.22, 0.09) | **0.10 (0.02, 0.19)** | 0.06 |
| Subscapular | 0.07 (-0.01, 0.15) | -0.03 (-0.19, 0.13) | 0.10 (0.01, 0.19) | 0.17 |
|  |  |  |  |  |
| OPE metabolite concentrations were log2-transformed and models were adjusted for maternal age at delivery, income, pre-pregnancy BMI, parity, infant sex and birth weight (note: models for infant weight were not adjusted for birth weight). Birth measurements were calculated as rates of change per week. Sex-specific models included an interaction term (exposure*sex). **Bolded** values indicate statistical significance based on 95% confidence intervals or p-for-EM ≤ 0.05. | | | | |

**Table S4.** Linear mixed effects models for repeated measurements of infant anthropometrics at birth and six weeks postpartum.

| **Anthropometric Measurement** | **Beta (95% CI)** | | |  |
| --- | --- | --- | --- | --- |
| **BCEP** | **Overall** | **Females** | **Males** | **p-for-EM** |
| Weight (kg) | 0.03 (-0.04, 0.10) | 0.02 (-0.11, 0.14) | 0.04 (-0.04, 0.12) | 0.77 |
| Length (cm) | -0.19 (-0.48, 0.09) | -0.24 (-0.79, 0.31) | -0.16 (-0.49, 0.16) | 0.82 |
| Abdominal circumference (cm) | -0.08 (-0.40, 0.24) | -0.07 (-0.73, 0.59) | -0.09 (-0.33, 0.15) | 0.96 |
| Head circumference (cm) | -0.08 (-0.30, 0.13) | -0.15 (-0.54, 0.25) | -0.04 (-0.27, 0.19) | 0.66 |
| Skinfold thickness (mm) |  |  |  |  |
| Iliac | -0.01 (-0.13, 0.11) | 0.04 (-0.13, 0.20) | -0.06 (-0.19, 0.07) | 0.34 |
| Thigh | **0.34 (0.16, 0.52)** | **0.34 (0.05, 0.63)** | **0.35 (0.12, 0.58)** | 0.96 |
| Tricep | 0.01 (-0.13, 0.14) | -0.07 (-0.25, 0.12) | 0.07 (-0.07, 0.21) | 0.26 |
| Subscapular | 0.02 (-0.13, 0.18) | -0.12 (-0.34, 0.10) | **0.14 (0.00, 0.28)** | **0.05** |
|  |  |  |  |  |
| **BDCPP** | **Overall** | **Females** | **Males** | **p-for-EM** |
| Weight (kg) | 0.06 (-0.03, 0.14) | -0.05 (-0.18, 0.07) | **0.14 (0.03, 0.24)** | **0.02** |
| Length (cm) | **0.44 (0.01, 0.87)** | 0.46 (-0.31, 1.23) | 0.43 (-0.12, 0.98) | 0.95 |
| Abdominal circumference (cm) | 0.25 (-0.18, 0.68) | 0.09 (-0.70, 0.88) | 0.37 (-0.10, 0.84) | 0.54 |
| Head circumference (cm) | -0.02 (-0.25, 0.21) | -0.33 (-0.72, 0.06) | 0.21 (-0.05, 0.47) | **0.04** |
| Skinfold thickness (mm) |  |  |  |  |
| Iliac | 0.07 (-0.04, 0.18) | 0.08 (-0.09, 0.26) | 0.06 (-0.08, 0.21) | 0.87 |
| Thigh | 0.22 (-0.01, 0.46) | 0.16 (-0.23, 0.56) | 0.27 (-0.02, 0.57) | 0.65 |
| Tricep | -0.11 (-0.25, 0.03) | **-0.24 (-0.48, 0.00)** | -0.02 (-0.18, 0.15) | 0.13 |
| Subscapular | 0.01 (-0.26, 0.27) | -0.15 (-0.57, 0.28) | 0.12 (-0.20, 0.45) | 0.29 |
|  |  |  |  |  |
| **DPHP** | **Overall** | **Females** | **Males** | **p-for-EM** |
| Weight (kg) | 0.01 (-0.09, 0.11) | **-0.19 (-0.36, -0.02)** | 0.07 (-0.04, 0.18) | **0.02** |
| Length (cm) | 0.05 (-0.45, 0.55) | -0.24 (-1.28, 0.81) | 0.13 (-0.45, 0.72) | 0.54 |
| Abdominal circumference (cm) | **-0.5 (-0.86, -0.14)** | **-0.88 (-1.74, -0.02)** | **-0.38 (-0.75, -0.01)** | 0.30 |
| Head circumference (cm) | -0.09 (-0.42, 0.23) | -0.59 (-1.27, 0.10) | 0.05 (-0.32, 0.43) | 0.11 |
| Skinfold thickness (mm) |  |  |  |  |
| Iliac | -0.02 (-0.21, 0.18) | -0.09 (-0.52, 0.33) | 0.02 (-0.20, 0.24) | 0.64 |
| Thigh | 0.06 (-0.31, 0.43) | -0.10 (-0.68, 0.48) | 0.12 (-0.33, 0.57) | 0.55 |
| Tricep | -0.08 (-0.31, 0.15) | 0.10 (-0.27, 0.47) | -0.15 (-0.42, 0.13) | 0.28 |
| Subscapular | -0.11 (-0.36, 0.15) | -0.27 (-0.72, 0.18) | -0.05 (-0.33, 0.23) | 0.39 |

All measurements were made at birth (mean±SD = 1.4±1.2 days) and six weeks postpartum (mean±SD = 6.8±1.1 weeks). OPE metabolite concentrations were log_2_-transformed and models were adjusted for maternal age at delivery, income, pre-pregnancy BMI, parity, infant sex, and age at the time of six-week anthropometric measurements. Overall and sex-specific effect estimates and 95% confidence intervals are presented since some interaction terms (exposure*sex) indicated effect modification by infant sex (p-for-EM ≤ 0.05). **Bolded** values indicate statistical significance based on 95% confidence intervals or p-for-EM ≤ 0.05. Results correspond to those presented in **Figure 1**.

| **Table S5.** Baby Eating Behavior Questionnaire (BEBQ) assessment of infant feeding behavior. | | | |  |
| --- | --- | --- | --- | --- |
| **BEBQ Trait** | **OPE (Beta (95% CI))** | | | |
|  | **BCEP** | **BDCPP** | **DPHP** | |
| General Appetite | 0.11 (-0.04, 0.27) | -0.002 (-0.24, 0.24) | 0.00 (-0.25, 0.25) | |
| Enjoyment of Food | 0.05 (-0.05, 0.15) | 0.06 (-0.10, 0.21) | -0.01 (-0.17, 0.15) | |
| Food Responsiveness | -0.02 (-0.14, 0.10) | **0.23 (0.06, 0.40)** | 0.03 (-0.17, 0.22) | |
| Slowness in Eating | -0.005 (-0.14, 0.13) | -0.12 (-0.33, 0.08) | -0.19 (-0.40, 0.02) | |
| Satiety Responsiveness | 0.003 (-0.10, 0.11) | -0.06 (-0.22, 0.10) | 0.03 (-0.14, 0.19) | |

Baby Eating Behavior Questionnaire (BEBQ) responses about infant feeding behavior provided by mothers at approximately six weeks post-partum. OPE metabolite concentrations were log_2_-transformed and multiple linear regression models were adjusted for maternal age at delivery, income, pre-pregnancy BMI, parity, and infant sex. **Bolded** values indicate statistical significance based on 95% confidence intervals. Results correspond to those presented in **Figure 2**.
